# Leading through crisis: care home managers’ COVID-19 experiences examined through systems-led setbacks and social positioning theory

**DOI:** 10.3389/fpubh.2026.1916166

**Published:** 2026-09-10

**Authors:** Fiona Marshall, Adam Gordon

**Affiliations:** 1 School of Health Sciences, Queen’s Medical Centre, University of Nottingham Medical School, Nottingham, United Kingdom; 2 Academic Centre for Healthy Ageing, Barts Health NHS Trust, Whipps Cross Hospital, London, United Kingdom; 3 Wolfson Institute of Population Health, Queen Mary University of London, London, United Kingdom; 4 NIHR Healthtech Research Centre for Community Healthcare, Oxford, United Kingdom

**Keywords:** care homes, governance, healthcare organisation, pandemic, policy, social positioning theory, systems-led setbacks

## Abstract

**Introduction:**

Care homes in England experienced disproportionate harm during the COVID-19 pandemic, yet existing policy analyses offer limited explanation of the structural and positional vulnerabilities faced by care home managers. This conceptual paper applies systems-led setbacks and social positioning theory to examine how pandemic policies shaped care home management across macro, meso and micro contexts.

**Methods:**

Qualitative research evidence relating to care home management during the pandemic was examined using a multi-level thematic analysis. Evidence was organised across six policy domains: hospital discharge, communication with families, personal protective equipment, end-of-life care, testing and surveillance, and vaccine roll-out. Recurring patterns were interpreted through the combined theoretical framework.

**Results:**

Five overarching themes were identified: liminal positioning, accountability displacement, rights erosion, information asymmetries and temporal misalignments. Across all six domains, managers were required to implement complex and frequently contradictory directives while operating with limited authority, incomplete information and insufficient institutional support. System-level decisions therefore interacted with managers’ marginal organisational position, amplifying risk for both staff and residents.

**Conclusion:**

Systems-led setbacks and social positioning theory provide greater explanatory depth than conventional policy analysis by showing how structural inequities and positional vulnerabilities compounded pandemic harms. Future preparedness frameworks should explicitly recognise the authority, information needs and institutional position of care home leaders, and involve them directly in policy design and implementation to reduce avoidable harm to people living and working in care homes.

## 1 Introduction

The COVID-19 pandemic exposed structural weaknesses in governance, coordination, communication and resource allocation across health and social care systems worldwide. Few sectors experienced these vulnerabilities more acutely than long-term care homes, which experienced high mortality rates, marked service disruption and organisational strain during successive waves of infection (1). Many of the difficulties encountered by care home providers arose not solely from the virus itself but from the ways in which uncertainty was transmitted and managed across interconnected health and social care systems.

During the COVID-19 pandemic, scientific knowledge evolved rapidly. Testing technologies were developed and deployed in real time (2), public health guidance changed frequently (3) and policymakers were required to make consequential decisions using incomplete information (4). Health and social care organisations faced unprecedented pressures relating to workforce capacity, infection control, service continuity and resource availability. These circumstances created disruptions arising from incomplete knowledge, changing expectations, inconsistent information flows and unpredictable resource availability.

Across Europe and other high-income countries, care homes provide residential support, personal care and some healthcare services for older adults with complex health and social care needs (5). Residents frequently experience multimorbidity, frailty, cognitive impairment and functional dependency (6), making them particularly vulnerable to adverse outcomes during public health emergencies.

Despite their importance, care homes have historically occupied an ambiguous position within health and social care systems. In England, the duty of care for care home residents is complex and distributed across multiple organisations. Healthcare services are primarily funded through the National Health Service (NHS), whereas social care provision operates through a mixed economy involving local authorities, independent providers and private funding arrangements. This fragmentation has contributed to longstanding concerns regarding coordination, information sharing, workforce development and strategic integration between health and social care sectors (7). Prior to the pandemic, numerous reports highlighted challenges facing the care-home sector, including workforce shortages, difficulties recruiting and retaining staff, financial pressures, increasing resident complexity and limited access to specialist healthcare support (8). From a systems perspective, these pre-existing vulnerabilities are important because they influence how organisations respond to external shocks. Systems characterised by fragmented governance arrangements, multiple accountability structures and unequal resource distribution may be particularly susceptible to disruption during periods of crisis (9).

The emergence of COVID-19 in early 2020 posed unprecedented challenges for care homes internationally. Residents were disproportionately affected by severe disease and mortality (10), while providers faced the simultaneous demands of infection prevention, service continuity, workforce protection and resident wellbeing (11). In England, care homes experienced the highest mortality rates associated with the pandemic, prompting widespread concern regarding preparedness and system resilience (12).

A growing body of research has documented the experiences of care-home providers during the pandemic. Commonly reported challenges included difficulties accessing PPE (13), uncertainty surrounding testing procedures (14), rapidly changing infection-control guidance (3), restrictions on family visiting (15), pressures associated with hospital discharge policies (16) and the complexities of vaccine implementation (17, 18). Viewed collectively, these issues highlight broader systemic dynamics that influenced care homes’ ability to respond effectively during the crisis.

Research undertaken during the pandemic highlighted the critical role of care-home managers in navigating these challenges (3, 13). Managers frequently acted as intermediaries between national policy directives, regional organisations, healthcare providers, residents, families and staff. Their responsibilities expanded considerably as they interpreted changing guidance, coordinated infection-control measures, communicated with families, supported staff wellbeing and maintained service delivery under exceptionally challenging circumstances.

Several studies have emphasised the emotional and operational pressures associated with these responsibilities (19). Care-home managers reported experiences of moral distress, exhaustion, frustration and professional isolation. However, while such accounts provide important insights into the lived realities of pandemic leadership, they do not fully explain why particular challenges emerged repeatedly across multiple domains of pandemic response.

This paper argues that many of the challenges experienced by care-home managers can be understood as manifestations of systems-led setbacks (20). Systems-led setbacks are defined here as structural constraints originating within policy, governance, coordination or resource-allocation processes that undermine the ability of organisations and individuals to fulfil their responsibilities. Unlike explanations that focus on individual errors or local operational failures, systems-led setbacks draw attention to the ways in which broader organisational arrangements can inadvertently generate adverse consequences for those at the frontline. Such setbacks are particularly likely to emerge during periods of uncertainty, when rapidly evolving information, competing priorities and fragmented decision-making create conditions in which responsibilities become detached from the resources, authority and support necessary for their fulfilment.

To examine these dynamics, this paper draws upon longitudinal research undertaken with care-home managers in England during the COVID-19 pandemic (3, 13). The study focuses on six domains that repeatedly emerged within managers’ accounts: Personal Protective Equipment (PPE) provision, hospital discharge procedures, end-of-life care, family communication and visiting, testing and surveillance, and vaccine rollout. Although these domains appear distinct, they reveal common patterns relating to uncertainty, organisational dependence and responsibility-capacity mismatches across multiple levels of the health and social care system.

The analysis is informed by Social Positioning Theory (SPT), a framework derived from Cambridge Social Ontology that examines how rights, obligations and practises are distributed across social systems (21). SPT is particularly useful for understanding how organisational actors become positioned within networks of responsibility and authority, and how these positions influence their capacity to respond during periods of disruption. Applied to the pandemic context, the theory provides a means of examining how care-home managers were positioned within broader systems of governance and how these positions shaped their experiences of uncertainty and constraint.

A growing body of literature suggests that uncertainty represents a defining characteristic of major crises (22). During public health emergencies, decision-makers frequently operate with incomplete information regarding disease transmission, intervention effectiveness, resource availability and future developments. Under such conditions, organisations must make consequential decisions while simultaneously adapting to rapidly changing circumstances. Emerging consensus from the UK COVID Inquiry has determined that earlier, more decisive responses around the COVID-19 pandemic amongst policymakers, could have prevented deaths (23). Modelling suggests, for example, that 23,000 lives could have been saved if lockdown had occurred 1 week earlier (23).

Uncertainty is often conceptualised as a problem of knowledge deficits. However, organisational research suggests that uncertainty also reflects relationships between information, authority, resources and decision-making structures (24). The effects of uncertainty depend not only on what is known or unknown but also on how information is distributed, interpreted and acted upon across organisational systems.

The paper advances three central arguments. First, many of the difficulties experienced by English care homes during the pandemic were manifestations of broader uncertainty cascading through health and social care systems. Second, uncertainty frequently generated systems-led setbacks by creating mismatches between organisational responsibilities and the resources, authority or information required to fulfil them. Third, social positioning theory provides a valuable framework for understanding why these effects were experienced so intensely within the care-home sector and how similar vulnerabilities may emerge during future crises.

## 2 Methods

Before outlining the methods, we present the overarching theoretical frameworks of Systems Led Setbacks and Social Positioninig theory.

### 2.1 Systems-led setbacks

Systems-led setbacks are structural impediments arising from governance arrangements, policy decisions, organisational processes or resource-allocation systems that undermine the ability of actors to meet expected obligations (20). They originate beyond the immediate control of those experiencing their effects and are best understood as properties of systems rather than individuals.

Four recurring mechanisms characterise systems-led setbacks in the pandemic context. *Contradictory expectations* arose where guidance from different parts of the system was incomplete, inconsistent or difficult to reconcile with operational realities, requiring managers to navigate competing obligations without clear mechanisms for resolution. *Resource–responsibility mismatches* occurred where managers remained accountable for infection prevention, staff protection and resident wellbeing yet lacked reliable access to PPE, testing capacity, staffing support or timely information. *Information asymmetries* reflected broader patterns in the distribution of knowledge—not merely communication problems—where information relating to testing, hospital discharges, infection risks and policy changes was delayed, incomplete or inconsistently communicated. *Temporal misalignment* arose from differences between policy timescales and implementation timescales: national guidance could change rapidly, whereas operational implementation required time for communication, interpretation, planning and delivery.

Together, these mechanisms illustrate how uncertainty becomes embedded within organisational processes and is subsequently experienced as systems-led setbacks by frontline actors.

### 2.2 Social positioning theory

Social Positioning Theory (SPT), developed within Cambridge Social Ontology, conceptualises society as a network of interconnected positions (21). Positions are not simply organisational roles but locations within social systems that carry particular rights, obligations and associated practises. Social outcomes emerge through interactions between these positions and the relationships connecting them.

Within the pandemic context, three organisational levels were particularly important. *Macro-level positions*—government departments, regulatory agencies, national public health bodies—possessed significant authority to establish priorities, allocate resources and define expectations. *Meso-level positions*—regional organisations, commissioning bodies, health and care providers, local authorities—were responsible for translating national policy into operational practise and shaped how uncertainty was communicated and managed. *Micro-level positions*—care-home managers, staff, residents and families—carried substantial operational responsibilities but often possessed limited influence over the policy and organisational processes shaping their work.

These levels are not intended to imply simple hierarchy; rather, they highlight interdependencies through which decisions, information and resources moved through organisational systems.

### 2.3 Integrating systems-led setbacks and social positioning theory

Integrating systems-led setbacks into SPT enhances the framework’s analytical power in four ways. It provides vocabulary for *structural causation*, analysing how conditions at higher levels causally shape outcomes at lower levels. It reveals *cascading accountability*, showing how accountability systems often invert causal chains by holding lower-level positions accountable for failures caused by higher-level structural conditions. It identifies *positional vulnerability*, where liminal positions—characterised by high obligations but limited rights—are particularly susceptible to systems-led setbacks. And it suggests *reform pathways*, indicating that addressing systems-led setbacks requires structural reforms at originating levels rather than exhortations to frontline actors. This integrated framework preserves SPT’s core insights while adding explanatory power for understanding crises and structural injustices.

### 2.4 Data sources

This study drwe on longitudinal qualitative research examining care-home managers’ experiences of the COVID-19 pandemic in England between 2020 and 2022 (3, 13), conducted during a period of exceptional organisational disruption. To situate participants’ accounts within the broader pandemic response, archived national policy and guidance data (using the COVID-19 tracker and governmental archives) were analysed across six critical domains of care-home policy and practise: PPE supply and allocation; hospital-to-care-home discharge procedures; end-of-life and palliative care provision; family communications and visiting restrictions; COVID-19 testing and infection surveillance; and vaccine prioritisation and distribution. These domains were selected to capture the intersection of care-home responses with wider healthcare system structures, enabling analysis of multi-level positional dynamics.

### 2.5 Analytical approach

Thematic analysis, as conceptualised by Braun and Clarke (25)—defined as “a method for identifying, analysing, and reporting patterns (themes) within data”—provided the methodological foundation. In line with Braun and Clarke’s distinction between inductive and deductive orientations, the analysis was theoretically driven, drawing on critical thematic analysis with social positioning theory (21) serving as the organising framework. Data were systematically coded across seven theoretical dimensions: social positions at macro, meso, and micro levels; associated rights; associated obligations; position-specific practises; processes of positional emergence or contestation; systems-led setbacks; and cascading effects across organisational hierarchies.

### 2.6 Analytical process

Following Braun and Clarke’s (25) six-phase process, the analysis proceeded as follows. In Phase 1 (Familiarisation), close reading of all documents generated initial notes identifying key positions, rights, obligations, and structural challenges. Phase 2 (Theoretical Coding) involved systematic deductive coding of each document through the constructs of social positioning theory, interrogating which positions were referenced or implied, what rights and obligations pertained, what practises were described or prescribed, and what structural failures were evident. Phase 3 (Theme Development) organised codes into preliminary themes representing recurring positional dynamics, systems-led setbacks, and cascading effects. Phase 4 (Theme Refinement) involved iterative review of themes against coded data to ensure internal coherence and external distinctiveness. Phase 5 (Theme Definition) established precise definitions specifying the positional dynamics, setbacks, and theoretical significance captured by each theme. Phase 6 (Synthesis) integrated the themes into a coherent analytical narrative demonstrating how social positioning theory, augmented by the concept of systems-led setbacks, illuminates COVID-19 care-home responses.

### 2.7 Analytical rigour

Rigour was upheld through three principal strategies consistent with Braun and Clarke’s quality criteria. Theoretical consistency ensured that all coding and theme development remained grounded in social positioning theory’s core constructs throughout. Systematic coverage required each document to be analysed across all theoretical dimensions, ensuring comprehensive application of the framework. Multi-level analysis maintained explicit attention to macro, meso, and micro positions, ensuring positional dynamics were captured across organisational hierarchies and that no level of analysis was inadvertently privileged.

### 2.8 Ethical approval

The interview components of this work were approved by the Faculty of Medicine & Health Sciences Research Ethics Committee, University of Nottingham-02-0420. All participants had mental capacity to provide written consent and provided written informed consent prior to participation in the study. All research procedures were conducted in keeping with the Declaration of Helsinki and Good Clinical Practise.

## 3 Results

As a precursor to more detailed analysis, the six domains of pandemic response were broken down into macro/meso/micro level responses. These are presented below with illustrative quotes to contextualise each domain from a care home manager’s perspective (Tables 1–6).

**Table 1:** PPE supplies and education.

Level	Key issues
Macro level [Governmental/National]	Government assurances of adequate PPE supply chains to care homes were undermined by commercial exploitation from suppliers, with no emergency pricing restrictions enacted. While a booking and reservation system was introduced to facilitate equitable distribution based on need, policy responses were largely punitive in nature, failing to account for the operational realities and contextual nuances of care home environments.
Meso level [Local Community]	Local distribution was characterised by fragmentation, with inequitable access particularly evident for rural and remote care homes. Collection hubs were established primarily in urban and urban-fringe areas, with NHS services receiving discretionary priority. Short pick-up windows and punitive responses to late collection, combined with a lack of provision for managers to escalate concerns and limited appreciation of workforce capacity constraints and staff travel challenges, further compounded access difficulties.
Micro level [Individual Care Homes]	Care homes were required to navigate newly established digital booking and reservation systems with limited flexibility, creating significant operational burden. Frustrations with supplier inflexibility prompted reliance on informal networks, including established inter-organisational relationships and social media appeals to local sectors (e.g., veterinary practises), to secure supplementary supplies. Uncertainty of supply contributed to elevated staff stress and engagement with an inadequate procurement system.
Consequences	The cumulative effect of these systemic failures was highly variable access to PPE, precipitating increased infection risk among staff due to repeated PPE use or inadequate quality and quantity. Care homes resorted to reliance on donations, improvised PPE fabrication (including adapted clothing and cloth masks by staff and residents), and informal sourcing. Professional and ethical pressures associated with sub-optimal barrier protection were compounded by the near absence of enhanced infection control training for the sector. Inflated pricing of core PPE items—including hand sanitisers, masks, and linen—created cost-driven inequity in access, prioritising financial capacity over clinical need.

*“I turned up 20 min late to pick up supplies, but our supplies had been given to an NHS community nurse, who had arrived in desperate need. We had to book and pick up yet this nurse was just given the masks. There wasn’t enough to go round. I wanted to scream.”* Manager, April 2020.

**Table 2:** Discharge procedures, testing and hospital discharge.

Level	Key issues
Macro level [Governmental/National]	Government assurances that all patients discharged from hospital would be tested prior to transfer, and that care homes would not be pressured into accepting patients without results, were not consistently upheld. Whilst clear guidance was issued to hospital discharge teams and clinicians, the corresponding guidance for the care home sector was widely regarded as ambiguous and excessively lengthy. The planned utilisation of Nightingale hospitals to accommodate patients awaiting test results—thereby relieving pressure on acute beds—did not materialise.
Meso level [Local Community]	Discharge and testing procedures at the local level were characterised by fragmentation and inconsistent adherence to guidance by NHS staff, with some care homes subject to pressure to accept patients in the absence of confirmed test results. Oversight of which homes possessed appropriate physical capacity for effective zoning and isolation of discharged patients was neither comprehensive nor timely. There were instances of unannounced nighttime discharges, and limited understanding of the diversity, contextual knowledge, and specialist expertise of local community care home provision.
Micro level [Individual Care Homes]	At the care home level, effective discharge management was contingent upon established working relationships between named hospital discharge teams and individual managers, introducing significant variability in practise. Operational challenges included poorly timed discharges and insufficient staffing capacity to accommodate new arrivals safely. The absence of on-site rapid testing precluded prompt screening of incoming residents or those presenting with initial symptoms. Care home managers faced duplication of effort through reporting bed status, isolation capacity, and staffing data to multiple agencies, whilst instances of their professional judgement being overridden generated frustration and undermined safe care provision. Discharge pathways from other residential settings, private homes, and recent overseas arrivals lacked codified procedural guidance.
Consequences	The cumulative impact of these failures posed material risks to care home safety, creating conditions conducive to the introduction and spread of infection from hospital settings. Communication breakdowns between managers and discharge teams eroded supportive dialogue, whilst uncertainty and inadequate information-sharing between local NHS teams and care homes generated considerable stress. Care home managers reported feeling unheard. Administrative duplication imposed substantial demands on already stretched staffing and time resources. Delayed and inaccurate communication of test results—including instances of lost results and unnecessary isolation of asymptomatic, test-negative residents—further undermined effective infection control and resource management.

*“I told them [hospital discharge team] we could not accept a patient without the test result, and we had no isolation beds left…just before midnight the ambulance arrived, no notice given with a 92-year-old man. The night staff could not refuse him, and the ambulance staff were sorry. But he proved positive 30 odd hours later and by the end of the week it was rife in the home. The hospital did not comply”* Manager, June 2020.

**Table 3:** Provision of end-of-life care.

Level	Key issues
Macro level [Governmental/National]	Government guidance directed at care home staff on end-of-life care was limited in scope, as was access to specialist expertise. Essential medications and equipment, including oxygen, were prioritised for NHS settings. Despite mounting mortality within the sector, the government persisted in pressing care homes to accept new residents from the NHS, whilst delaying consultation with key sector experts—including geriatricians and hospice specialists—regarding end-of-life resource needs and the unique challenges of care provision in this setting. Accurate data on the scale and trajectory of care home deaths was absent, and policy responses continued to rely on punitive compliance mechanisms with little recognition of the contextual complexities of the sector.
Meso level [Local Community]	Local provision of essential medications, equipment, and clinical advice to care homes experiencing elevated mortality was fragmented and inconsistent. Care homes that implemented early lockdowns faced punitive regulatory breach threats, whilst duplication in death notification procedures consumed scarce staff time and capacity. There was a broader failure to appreciate the diversity, contextual knowledge, and specialist expertise of local community care home provision. Atypical presentations of infection among residents were dismissed, contributing to treatment delays and potentially increased mortality.
Micro level [Individual Care Homes]	In the absence of adequate systemic support, care homes relied on established professional relationships with pharmacists, hospice staff, and general practitioners to circumvent NHS allocation systems and secure necessary practical and clinical resources. Staff assumed extended roles, including determination of death, administration of intravenous fluids, and close monitoring of residents at end of life. The immense challenges of delivering dignified end-of-life care under these conditions compounded professional uncertainty, exacted a significant emotional toll on staff, and disrupted routine care home operations. Profound professional and personal ethical dilemmas resulted in employee attrition and long-term sickness absence.
Consequences	These conditions compromised the dignity of dying for residents and undermined the professional and personal ethical standards of care home staff, who reported concern that care provided was sub-optimal. The experience confirmed, among care home managers, longstanding perceptions of entrenched misunderstanding and negative assumptions about the sector held by decision-makers at both national and local levels. Administrative duplication in reporting to authorities placed further demands on already depleted staffing and time resources. Data on care home deaths and bed capacities across the sector remained delayed and inaccurate throughout.

*“We tried our best but its simply fighting and more fighting. Many died without family, alone and we could do nothing as they tried to breathe, to not have pain and a dignified death. It went against all I practise and this still haunts me. Our residents are our family. It was just so unfair, all of it….”* Manager, August 2020.

**Table 4:** Family communications and visits.

Level	Key issues
Macro level [Governmental/National]	National guidance governing visits by adult relatives was extensive and difficult to assimilate into practise, prescribing restrictions on who could visit, when, and where. Multiple iterations were issued from July 2020 onwards, incorporating measures including outdoor (garden) visits, window communications, a single-visitor limit, and mandatory PPE use. The designated “essential caregiver” role, permitting sustained access by a named relative, was not introduced until 2021.
Meso level [Local Community]	Local authorities provided fragmented and inconsistent clarification to care homes, with no standardised information materials produced for families. There was limited appreciation of the needs of residents with dementia and the practical implications of enforcing visiting restrictions on this population, including those approaching end of life. Strict interpretation of national guidance generated regional and local discrepancies, with some evidence of inconsistency with national policy. Financial support for physical modifications to enable safe visitor isolation was minimal, and the workforce capacity required to oversee visits was not adequately understood at the local level.
Micro level [Individual Care Homes]	Chronic understaffing created considerable operational pressure in accommodating visitors safely without adequate supervision or support for residents and relatives. Staff were required to communicate frequently changing and ambiguous guidance, compounding difficulties in conveying accurate information to both colleagues and families. Enforcement of strict measures—including testing, designated visiting zones, PPE use, window observations, and end-of-life restrictions—placed substantial demands on care home staff. Reliance on digital and telephone communications proved sub-optimal, particularly for older relatives and family members. Some homes imposed total bans on visits, with certain restrictions persisting until 2022 (39). The discretionary application of the recommended “key relative” scheme from January 2021 introduced further inconsistency across the sector. Cumulatively, these pressures generated frustration and exhaustion among care home staff.
Consequences	Variable interpretation of guidance resulted in inconsistent messaging and adherence within and across homes, complicating risk assessment in relation to visitor access. Visiting restrictions exacerbated deconditioning and deterioration among residents with dementia, compounding social isolation and loneliness, particularly during quarantine periods. Blanket bans heightened distress and suspicion among relatives and severed the practical and emotional care that families—especially those of residents with dementia—provide. Staff reported significant professional and ethical pressures, encompassing concerns around human rights, missed end-of-life visits, emotional costs to families, and accelerating deconditioning among vulnerable residents. Reliance on donated materials to adapt visiting spaces further underlined resource constraints. At a broader level, variable and restrictive practises generated reputational damage at both local and national levels, prompting legal challenges and calls for legislative reform to protect the right to essential visits by relatives.

*“We were portrayed as not caring, but I worried about spouses having to manage on their own throughout all this. They could not see their loved ones, except if end-of-life. It was so unfair. Masked relatives were not recognised by residents, and many were utterly bewildered by our attempts at screentime sessions. The mental health of families suffered and we could do nothing. It was so cruel. The sudden lockdown over Christmas was the final straw in my eyes.”* Manager, January 2021.

**Table 5:** Testing for Covid infection.

Level	Key issues
Macro level [Governmental/National]	The national testing strategy for care homes underwent several significant policy shifts. Following the cessation of care home testing in March 2020, asymptomatic testing was introduced from April 2020, with mandatory testing of staff and residents enacted from 8 June 2020. A research programme was initiated in July 2020 to quantify the spread and levels of infection across 100 care homes. Lateral flow tests were confirmed as accurate for use in care home settings from late December 2020, though this remained subject to ongoing academic debate well into 2021. No payment was initially available for staff complying with ten-day self-isolation requirements, with financial support only introduced from June 2020. Guidance was issued on care home admissions and pre-admission testing, alongside support for local authorities to manage the spread of infection.
Meso level [Local Community]	The requirement to test hospital patients prior to transfer to care homes was not consistently adhered to, and difficulties with testing data, outcomes, and the track and trace application were widely reported. Distribution of test kits was variable, with smaller and more rural care homes particularly disadvantaged in terms of both delivery and collection. Tracking of agency and cross-site staff presented additional challenges. Pressure was exerted on care homes to accept untested patients from hospital settings. Lateral flow tests were distributed to local authorities from November 2020.
Micro level [Individual Care Homes]	Securing adequate test samples from residents with cognitive impairment or other individual factors presented practical difficulties. Maintaining isolation of test-positive residents was complicated by building configurations, behavioural and cognitive challenges, and elevated risk of harm. Testing timetables operating within the working week excluded staff employed on weekend-only contracts, whilst support for the management of positive results was unavailable during evenings and weekends. Considerable anger and scepticism were directed at both the mandate for testing and the efficacy of the system overall, encompassing the decision to cease testing in March 2020, site accessibility and booking mechanisms, the track and trace application, and test kit logistics. Staffing pressures in some homes led to staff being informally pressured to remain at work.
Consequences	The testing regime generated heightened anxiety among staff, residents, and family members regarding the safety and accuracy of testing procedures and results. Substantive moral and professional uncertainty surrounded the mandate for staff testing. Tensions arose when staff were required to insist on testing among designated family visitors from December 2020. The process of booking tests, receiving timely results, and accessing self-isolation support payments was fragmented, raising concerns about the potential presence of unidentified positive carriers within care home settings. Instances of lost tests and results, alongside lengthy delays, further undermined confidence in the system. The absence of evening and weekend support for the management of positive test results represented a significant and unaddressed operational gap.

*“It did not really work well. Not only did we have lost tests, late results and difficulties with getting testing done. We also had to contend with fear, resistance and scepticism at the accuracy (of tests). Our staff were genuinely fearful that a positive would lead to a loss of pay, spread to their families and that they may have given it to others in the home. There was no way we could manage this with all the other stuff, but we had to. I must admit that I had to continue working from home with COVID, I had nobody else and had to manage the home. I went back in early and felt incredibly ill.”* Manager 14 December 2020.

**Table 6:** Vaccine roll-out from early December 2020.

Level	Key issues
Macro level [Governmental/National]	Fifty centralised hospital hub vaccination centres were established across the UK from 8 December 2020, with care home residents prioritised for vaccination from 30 December 2020. Vaccination was mandated for all care home staff from the same date, in the absence of an equivalent mandate for NHS staff—a distinction that generated considerable tension within the sector. Guidance for families required to provide consent for a family member’s vaccination was limited, as was guidance for local communities tasked with organising vaccine delivery. Rollout was intended to be delivered in collaboration with NHS community trust staff.
Meso level [Local Community]	Physical access for care home residents to hospital hub vaccination sites was minimal from the outset (8 December 2020), and no funding was provided to support the transportation of residents to these locations. There was reluctance at the local level to administer vaccines directly within individual care homes. Advice regarding acceptable consent processes for resident vaccination was variable, and guidance issued to local authorities (public health) and NHS teams on the practical organisation of vaccine delivery to the most vulnerable was limited.
Micro level [Individual Care Homes]	Care homes faced complex consent challenges, particularly regarding the most frail and vulnerable residents. Provision was unequal, with effective access often contingent on established working relationships between hub organisers and individual homes, introducing significant inequalities. The vaccine booking system was insufficiently robust and timely, with spatial inaccuracies affecting smaller towns and rural settings. The rollout was resource-intensive, coinciding with chronic staffing pressures and pandemic fatigue among care home staff. The mandating of vaccines for care home staff, in the absence of an equivalent requirement for NHS staff, generated anger and a strong sense of differential standards being applied across the sector.
Consequences	The rollout generated heightened anxiety about vaccine safety among staff, residents, and family members, alongside substantive moral and professional uncertainty regarding the mandate for staff vaccination. Staff reported tensions in being expected to encourage family vaccination whilst availability remained limited. The process of ordering and receiving vaccines was fragmented, generating operational difficulties. The inaccessibility of hospital hubs reinforced perceptions, widely held among care home managers, that the specific circumstances and needs of care home residents and staff were not adequately understood at national or local levels. Guidance for care home and NHS staff and local authorities to manage rollout among vulnerable residents was insufficient, and the absence of a robust reporting system for vaccine receipt was viewed as a further indicator of the systemic marginalisation of the care home sector.

*“The process of obtaining consent for vaccinations was difficult, with many residents uncertain and family members already stressed, having to make these decisions. Families wanted to know the risks and frankly we did not really know. Some staff hated the mandating of the vaccines and one left as she was too scared to have it. But it was a tough one all round.”* Manager, February 2021.

### 3.1 Discharge procedures, testing and hospital discharge

#### 3.1.1 Consequences

Variable interpretation of guidance leading to issues with consistent messaging and adherence.

#### 3.1.2 Social positioning theory analysis of care home experiences: cross cutting themes

Tables 7–12 map each of the six analysed domains to social positioning theory constructs, revealing how positions, rights, obligations, practises, emergence processes, systems-led setbacks, and cascading effects operated across domains.

**Table 7:** Personal protective equipment (PPE).

Dimension	Analysis
Social positions	Macro: DHSC managing national PPE supply, PHE defining PPE guidance and usage standards, NHS Supply Chain controlling distribution. Meso: Local authorities, CCGs, and regional procurement networks attempting to secure PPE for care homes, mutual aid networks emerging among care providers. Micro: Care home managers sourcing PPE through multiple channels, care staff using inadequate or improvised protection, residents receiving care from inadequately protected staff.
Rights	Macro positions: Right to define PPE standards, control national supply, prioritise allocation. Meso positions: Right to access national supply chains, receive allocation information, coordinate local procurement. Micro positions: Right to receive adequate PPE for safe care provision, refuse unsafe work, access same protection as NHS staff.
Obligations	Macro positions: Obligation to ensure adequate PPE supply, equitable distribution, clear usage guidance. Meso positions: Obligation to distribute PPE fairly, support care homes in procurement, coordinate regional supply. Micro positions: Obligation to provide safe working conditions, implement infection control, protect staff and residents.
Practises	Macro: Supply chain management, allocation decisions, guidance updates, procurement contracts. Meso: Regional distribution, local procurement coordination, mutual aid organisation. Micro: PPE ordering through multiple channels, stock management, usage training, improvised protection (bin bags, homemade masks), rationing supplies.
Emergence	PPE crisis repositioned care homes from routine supply chain participants to desperate competitors in scarce markets. Care home manager positions gained obligations to secure PPE through any means while losing rights to reliable supply. Care staff positions gained heightened infection risks while losing rights to adequate protection. Macro positions maintained control over allocation while deflecting accountability for shortages.
Systems-led setbacks	Supply Chain Failure: Macro-level procurement failures and NHS prioritisation left care homes without access to national supply chains. Guidance-Reality Mismatch: PHE guidance assumed PPE availability that did not exist, creating impossible compliance demands. Market Dysfunction: Care homes forced into unregulated markets faced with price gouging and fraud without macro-level intervention. Information Gaps: Lack of transparency about supply levels and allocation criteria prevented effective planning.
Cascading effects	Macro-level supply failures cascaded to meso-level scrambling for alternative sources, cascading to micro-level impossible choices between providing care without protection or refusing care. Guidance-reality mismatch cascaded to compliance failures, cascading to regulatory criticism of care homes for not following impossible standards. Market dysfunction cascaded to financial strain, cascading to some care homes unable to afford available PPE. Result: staff infections and deaths, care provision without adequate protection, moral injury from forced risk-taking, financial exploitation of desperate care homes, and erosion of trust in governmental positions.

**Table 8:** Discharge.

Dimension	Analysis
Social positions	Macro: Department of Health and Social Care (DHSC), NHS England, Public Health England (PHE) defining discharge policy and infection control standards. Meso: Hospital discharge coordinators, Clinical Commissioning Groups (CCGs), local authority adult social care teams mediating between national policy and local implementation. Micro: Care home managers deciding admission acceptance, care home staff implementing infection control, receiving residents with unknown infection status.
Rights	Macro positions: Right to define discharge criteria, set infection control standards, allocate resources. Meso positions: Right to receive clear guidance, access testing resources, coordinate across sectors. Micro positions: Right to refuse unsafe admissions, receive infection status information, access PPE and testing, receive clinical support for complex residents.
Obligations	Macro positions: Obligation to provide clear guidance, ensure testing availability, protect care home residents, support NHS capacity. Meso positions: Obligation to coordinate safe discharges, communicate between hospitals and care homes, ensure resource access. Micro positions: Obligation to accept appropriate admissions, implement infection control, provide safe care, protect existing residents.
Practises	Macro: Policy drafting, guidance issuance, resource allocation decisions. Meso: Discharge coordination meetings, capacity assessments, inter-organisational communication. Micro: Admission assessments, isolation protocols, infection control procedures, staff training, resident monitoring.
Emergence	Rapid repositioning as pandemic pressures mounted. “Discharge to Assess” policies repositioned care homes as NHS capacity relief valves. Care home manager positions gained new obligations (accept discharges to support NHS) without corresponding rights (refuse unsafe admissions, demand testing). Hospital discharge coordinator positions gained power to pressure care homes while infection control obligations remained ambiguous.
Systems-led setbacks	Contradictory Guidance: Simultaneous obligations to accept discharges (support NHS) and prevent infection entry (protect residents) with no resolution mechanism. Testing Unavailability: Macro-level failure to provide testing meant micro-level positions made admission decisions without infection status information. Resource Scarcity: PPE shortages meant care homes accepted potentially infected residents without adequate protective equipment. Temporal Pressure: Discharge timelines (24–48 h) misaligned with infection control requirements (14-day isolation), forcing impossible choices.
Cascading effects	Macro-level policy prioritising NHS capacity over care home safety cascaded to meso-level pressure on care homes to accept discharges, cascading to micro-level impossible situations where managers chose between abandoning NHS support obligations or endangering residents. Testing unavailability at macro level cascaded to information asymmetries at meso level, cascading to blind decision-making at micro level. Resource scarcity at macro level cascaded to tragic choices at micro level between self-protection and resident care. Result: widespread care home outbreaks, staff infections, resident deaths, and moral injury among frontline workers forced to accept known risks.

**Table 9:** End of life care.

Dimension	Analysis
Social positions	Macro: DHSC and NHS England defining palliative care policy and resource allocation, National Institute for Health and Care Excellence (NICE) issuing clinical guidance. Meso: CCGs commissioning palliative care services, hospices and specialist palliative care teams providing consultation, GPs prescribing and overseeing care. Micro: Care home managers coordinating end-of-life care, care staff providing direct palliative care (often without specialist training), residents dying in care homes rather than hospitals, families excluded from end-of-life presence.
Rights	Macro positions: Right to define palliative care standards, allocate specialist resources, issue clinical guidance. Meso positions: Right to access specialist consultation, prescribe medications, receive clinical support. Micro positions: Right to access specialist palliative care support, receive necessary medications and equipment, provide dignified deaths. Residents’ rights to pain management, comfort, family presence, and dignified death. Families’ rights to be present and participate in end-of-life care.
Obligations	Macro positions: Obligation to ensure equitable palliative care access, provide specialist support, supply necessary medications. Meso positions: Obligation to provide specialist consultation, prescribe appropriately, support care homes. Micro positions: Obligation to provide comfort and dignity, manage symptoms, facilitate family presence, honour advance care plans.
Practises	Macro: Palliative care policy development, resource allocation, guidance issuance. Meso: Specialist consultation (often remote), prescription of anticipatory medications, advance care planning. Micro: Symptom management, comfort care, family communication about deterioration, end-of-life visit facilitation, body care after death.
Emergence	Pandemic repositioned care homes as primary end-of-life care settings as hospital admissions declined. Care home staff positions gained obligations to provide complex palliative care without corresponding specialist training or support. Specialist palliative care positions became remote consultants rather than direct providers. Family positions lost rights to end-of-life presence, fundamentally altering death experiences.
Systems-led setbacks	Specialist Access Barriers: Macro-level failure to ensure specialist palliative care access meant care homes provided complex care without expert support. Medication Supply Issues: Anticipatory medication shortages and prescription delays meant inadequate symptom management. Training Gaps: No macro-level provision of palliative care training for care staff suddenly providing end-of-life care. Family Exclusion: Visiting restrictions applied even at end-of-life, with unclear guidance on exceptions, causing residents to die alone.
Cascading effects	Specialist access barriers at macro level cascaded to care homes attempting complex palliative care without support, cascading to inadequate symptom management and staff distress. Medication supply issues cascaded to residents experiencing preventable pain and distress. Training gaps cascaded to staff feeling unprepared and fearful of providing inadequate care. Family exclusion cascaded to profound grief and trauma, and staff bearing emotional burden of being sole presence at death. Result: residents dying in pain or distress, families traumatised by exclusion, staff moral injury from providing care they felt inadequate to deliver, and fundamental violations of dignified death principles.

**Table 10:** Family communications.

Dimension	Analysis
Social positions	Macro: DHSC and PHE defining visiting restrictions and infection control policies. Meso: Care Quality Commission (CQC) enforcing restrictions, local authority safeguarding teams monitoring resident wellbeing, advocacy organisations representing family interests. Micro: Care home managers implementing restrictions, care staff mediating family-resident contact, families positioned as infection risks rather than care partners, residents experiencing isolation.
Rights	Macro positions: Right to define public health restrictions, balance infection control and wellbeing. Meso positions: Right to enforce compliance, investigate safeguarding concerns, advocate for families. Micro positions: Care homes’ right to implement restrictions for infection control. Families contested rights to information, communication access, and physical presence. Residents’ rights to family contact, emotional wellbeing, and advocacy.
Obligations	Macro positions: Obligation to provide clear guidance balancing infection control and wellbeing, ensure communication alternatives. Meso positions: Obligation to enforce restrictions consistently, protect resident wellbeing, support families. Micro positions: Care homes obligated to prevent infection entry, maintain resident wellbeing, and facilitate family communication. Families obligated to comply with restrictions. Staff obligated to compensate for family absence.
Practises	Macro: Restriction policy development, guidance updates. Meso: Compliance monitoring, safeguarding investigations, advocacy campaigns. Micro: Visitor screening, window visits, video calls, increased staff-resident interaction, family communication via phone/email, and end-of-life visit facilitation.
Emergence	Visiting restrictions rapidly repositioned families from care partners to infection threats. Family positions lost rights (physical presence, direct care participation) while retaining obligations (emotional support, advocacy) without means to fulfil them. Care staff positions gained obligations to compensate for family absence (emotional support, communication facilitation) without additional resources or training. Resident positions became more dependent and isolated.
Systems-led setbacks	Contradictory Obligations: Care homes obligated to maintain resident wellbeing while preventing family contact, with extensive evidence that isolation harmed wellbeing. Resource Absence: No macro-level provision of communication technology, training, or staffing to support alternative contact methods. Guidance Ambiguity: Unclear criteria for “essential” visits created inconsistent implementation and family frustration. Accountability Displacement: Care homes held responsible for resident wellbeing deterioration caused by macro-level restriction policies.
Cascading effects	Macro-level blanket restrictions cascaded to meso-level enforcement pressure, cascading to micro-level impossible situations where care homes chose between infection control compliance and resident wellbeing. Resource absence at macro level cascaded to improvised solutions at micro level (staff personal devices for video calls, window visits in poor weather). Guidance ambiguity cascaded to inconsistent implementation, cascading to family perceptions of arbitrary decision-making and eroded trust. Result: resident cognitive and emotional decline, family trauma and exclusion, staff moral distress from witnessing harm, and breakdown of family-care home partnerships essential for quality care.

**Table 11:** Testing and surveillance.

Dimension	Analysis
Social positions	Macro: DHSC and PHE controlling testing policy and capacity, NHS Test and Trace managing testing infrastructure, Department prioritising testing allocation. Meso: Local public health teams coordinating outbreak response, laboratories processing tests, regional health protection teams investigating outbreaks. Micro: Care home managers requesting tests and managing outbreaks, care staff undergoing testing, residents tested for infection, symptomatic individuals isolated based on incomplete information.
Rights	Macro positions: Right to define testing priorities, allocate limited capacity, determine surveillance strategies. Meso positions: Right to access testing for outbreak investigation, receive timely results, coordinate public health response. Micro positions: Right to access testing for staff and residents, receive timely results, obtain public health support for outbreaks.
Obligations	Macro positions: Obligation to expand testing capacity, ensure equitable access, provide timely results, support outbreak management. Meso positions: Obligation to investigate outbreaks, provide public health guidance, coordinate testing. Micro positions: Obligation to identify infections, isolate cases, implement outbreak control, protect uninfected residents.
Practises	Macro: Testing policy development, capacity allocation, infrastructure development. Meso: Outbreak investigation, contact tracing, public health guidance provision. Micro: Symptom monitoring, test requesting, isolation implementation, outbreak management, infection control escalation.
Emergence	Testing scarcity repositioned care homes from routine surveillance participants to low-priority supplicants. Care home manager positions gained obligations to manage outbreaks without testing access needed for informed decisions. Macro positions-maintained control over testing allocation while care homes faced consequences of testing unavailability.
Systems-led setbacks	Testing Unavailability: Macro-level testing capacity failures meant care homes managed outbreaks without knowing infection status. Result Delays: When testing was available, delays (5–7 days) made results useless for outbreak control. Asymptomatic Transmission: Testing policies focused on symptomatic individuals despite evidence of asymptomatic transmission, creating false security. Surveillance Gaps: No systematic surveillance meant outbreaks detected only after significant spread.
Cascading effects	Testing unavailability at macro level cascaded to blind outbreak management at micro level, cascading to ineffective isolation decisions and continued transmission. Result delays cascaded to isolation decisions based on outdated information. Asymptomatic transmission combined with symptomatic-only testing cascaded to undetected spread. Surveillance gaps cascaded to late outbreak detection, cascading to larger outbreaks and more deaths. Result: preventable infections and deaths, ineffective outbreak control, staff and resident infections from undetected cases, and care homes blamed for outbreaks they lacked tools to prevent.

**Table 12:** Vaccine roll-out.

Dimension	Analysis
Social positions	Macro: DHSC and NHS England defining vaccine prioritisation and rollout strategy, Joint Committee on Vaccination and Immunisation (JCVI) providing clinical advice, NHS managing vaccine supply and distribution. Meso: CCGs and local vaccination services coordinating delivery, GPs and pharmacies administering vaccines, local authorities supporting care home access. Micro: Care home managers coordinating resident and staff vaccination, care staff deciding whether to accept vaccination, residents (or families) consenting to vaccination.
Rights	Macro positions: Right to define prioritisation, allocate supply, determine delivery models. Meso positions: Right to access vaccine supply, receive implementation guidance, coordinate local delivery. Micro positions: Right to priority access (care home residents in top priority group), receive clear information, access convenient vaccination, make informed decisions.
Obligations	Macro positions: Obligation to prioritise vulnerable populations, ensure equitable access, provide clear information, support implementation. Meso positions: Obligation to deliver vaccines to priority groups, provide accurate information, support informed consent. Micro positions: Obligation to facilitate resident and staff vaccination, provide information, support decision-making, maintain infection control until vaccination complete.
Practises	Macro: Prioritisation decisions, supply allocation, guidance development, communication campaigns. Meso: Vaccination clinic organisation, mobile vaccination teams, appointment scheduling, consent processes. Micro: Vaccination coordination, staff and resident information provision, consent facilitation, post-vaccination monitoring.
Emergence	Vaccine rollout repositioned care home residents as top priority group, recognising their vulnerability. However, care home manager positions gained complex coordination obligations without corresponding authority over staff vaccination decisions. Staff positions gained rights to refuse vaccination, creating tension with care home infection control obligations.
Systems-led setbacks	Implementation Delays: Despite top priority status, operational challenges delayed care home vaccination, with some residents waiting weeks while lower-priority groups were vaccinated. Guidance Changes: Frequent changes to prioritisation and delivery models created confusion and implementation challenges. Staff Hesitancy: No macro-level strategy to address staff vaccine hesitancy meant care homes managed this individually without resources. Supply Unpredictability: Uncertain supply and short notice for vaccination teams created logistical challenges.
Cascading effects	Implementation delays at macro level cascaded to care homes maintaining strict infection control longer than necessary, cascading to continued resident isolation and family exclusion. Guidance changes cascaded to repeated replanning at micro level, cascading to missed vaccination opportunities. Staff hesitancy without macro-level support cascaded to care homes attempting individual persuasion without expertise or resources. Supply unpredictability cascaded to last-minute coordination challenges, cascading to some residents missed in vaccination rounds. Result: delayed protection for vulnerable residents, continued outbreaks in unvaccinated care homes, staff-management tensions over vaccination, and inequitable access despite priority status.

### 3.2 Thematic findings across domains

Thematic analysis revealed five major themes that capture recurring patterns in how social positioning and systems-led setbacks shaped COVID-19 care home responses across the six domains. Each theme represents a distinct positional dynamic with characteristic systems-led setbacks and cascading effects.

#### 3.2.1 Theme 1: impossible obligations

*Definition*: Care home managers and staff occupied liminal positions characterised by high obligations but limited rights, creating systematic impossibilities where positioned actors were held responsible for outcomes, they lacked capacity to achieve.

*Positional Dynamics*: Across all six domains, care home positions were structured with extensive obligations—accept hospital discharges, maintain resident wellbeing despite family exclusion, provide safe care without adequate PPE, deliver complex palliative care, manage outbreaks without testing, coordinate vaccination—while lacking corresponding rights to refuse unsafe demands, access necessary resources, or receive adequate support. This liminal positioning created systematic position-capacity misalignments (40).

*Systems-Led Setbacks*: Liminal positioning was not accidental but resulted from macro-level policy decisions that assigned obligations downward without providing enabling conditions. Discharge policies obliged care homes to accept residents without providing testing. Visiting restrictions obligated care homes to maintain wellbeing without providing communication resources. PPE guidance obligated infection control without ensuring supply. This pattern represents a fundamental systems-led setback: structural design that systematically creates impossible situations for frontline positions.

##### 3.2.1.1 Illustrative examples

*Discharge Procedures*: Care home managers were obligated to accept hospital discharges to support NHS capacity while simultaneously obligated to protect existing residents from infection. Without access to testing, this created an impossible choice: refuse discharges and abandon NHS support obligations or accept discharges and risk resident safety. One manager described feeling “damned if you do, damned if you don’t” accepting discharges led to outbreaks and blame for inadequate infection control; refusing discharges led to pressure from hospitals and accusations of not supporting the NHS (13).

*PPE Provision*: Care staff were obligated to provide hands-on personal care while lacking adequate PPE. This created impossible situations where staff chose between self-protection (refusing care) and resident care (accepting infection risk). Many staff described moral injury from being forced to choose between their own safety and residents’ needs, with some continuing to provide care despite inadequate protection and subsequently becoming infected (13).

*Cascading Effects*: Liminal positioning cascaded through multiple pathways. Impossible obligations created moral injury as staff were forced into no-win situations (26). They generated operational failures as positioned actors lacked capacity to fulfil obligations. They produced accountability displacement as frontline actors were blamed for failures caused by structural positioning. They eroded trust as care homes lost confidence in governmental positions that assigned obligations without support.

*Theoretical Significance*: This theme demonstrates how social positioning theory illuminates structural injustice. Liminal positioning represents a systematic violation of the principle that obligations must be matched with enabling rights and resources. Systems-led setbacks transformed this positional vulnerability into operational crisis. The theme reveals that care home failures were not primarily due to individual inadequacy but structural positioning that created impossible situations (27, 28).

#### 3.2.2 Theme 2: accountability displacement and structural invisibility

*Definition*: Systematic patterns where accountability for failures was displaced downward from macro-level positions that caused problems to micro-level positions that experienced consequences, while structural causes remained invisible in dominant narratives. Positional Dynamics: Governmental positions at macro levels-maintained rights to define policy, allocate resources, and set standards while deflecting obligations to ensure implementation feasibility or provide necessary support. When policies failed, accountability was displaced to care home positions that lacked power to change structural conditions. This created a systematic pattern where those with least power over structural conditions bore greatest accountability for structural failures (41).

*Systems-Led Setbacks*: Accountability displacement was enabled by structural invisibility—the tendency for structural causes of failures to disappear from view while individual actions remained visible. When care homes experienced outbreaks after accepting untested discharges, the visible action (accepting discharge) was blamed while the structural cause (testing unavailability) remained invisible. This represents a meta-level systems-led setback: accountability systems that systematically obscure structural causation.

##### 3.2.2.1 Illustrative examples

*Testing and Surveillance*: Care homes were blamed for outbreaks and inadequate infection control despite lacking access to testing needed for effective outbreak management. Regulatory inspections criticised care homes for not isolating infected residents, without acknowledging that testing unavailability made it impossible to identify who was infected. The structural failure (macro-level testing capacity) remained invisible while the operational consequence (care home outbreaks) was attributed to care home inadequacy (23).

*Family Communications*: Care homes were held accountable for resident wellbeing deterioration (cognitive decline, depression, behavioural changes) caused by visiting restrictions mandated by macro-level policy. Families and regulators criticised care homes for not maintaining resident wellbeing, without acknowledging that wellbeing deterioration was a predictable consequence of isolation policies. The structural cause (blanket visiting restrictions) remained invisible while the consequence (resident decline) was attributed to inadequate care (23).

*Cascading Effects*: Accountability displacement cascaded through multiple mechanisms. It created defensive practises as care homes focused on documenting compliance rather than optimal care. It generated moral injury as frontline staff were blamed for failures beyond their control (26). It eroded trust between organisational levels as care homes felt scapegoated. It prevented learning as structural causes remained unaddressed while individual actors were blamed.

*Theoretical Significance*: This theme reveals how power operates through positioning. Macro-level positions-maintained power to define problems and assign blame while deflecting accountability for structural conditions they controlled. Social positioning theory with systems-led setbacks makes structural causation visible, revealing how accountability systems often invert causal chains. This has implications for organisational justice: fair accountability requires tracing failures to their structural origins rather than stopping at the most visible actors (34).

#### 3.2.3 Theme 3: rights erosion and obligation accumulation

*Definition*: Systematic patterns where care home positions lost rights while gaining obligations, creating increasingly untenable positional structures, while governmental positions-maintained rights while deflecting obligations.

*Positional Dynamics*: The pandemic involved asymmetric repositioning. Care home positions experienced erosion (lost rights to refuse unsafe admissions, access reliable supply chains, receive specialist support, demand accountability) while experiencing obligation accumulation (gained obligations to accept discharges, compensate for family absence, provide complex care, manage outbreaks) (45). Governmental positions-maintained rights (define policy, allocate resources, set standards) while deflecting obligations (ensure feasibility, provide support, accept accountability) (28).

*Systems-Led Setbacks*: This asymmetric repositioning represents a fundamental systems-led setback: structural changes that systematically disadvantage lower-level positions while protecting higher-level positions. Rights erosion without obligation reduction created position-capacity misalignments. Obligation accumulation without rights expansion created impossible demands. This pattern reveals how crisis can be exploited to restructure positions in ways that benefit powerful actors while disadvantaging vulnerable ones.

##### 3.2.3.1 Illustrative examples

*Discharge Procedures*: Care homes lost rights to refuse admissions (pressure to accept all discharges to support NHS) while gaining obligations to manage potentially infected residents without testing. This represented a fundamental restructuring of the care home position from autonomous decision-maker to NHS capacity relief valve, with obligations expanded but rights contracted (35).

*End-of-Life Care*: Care homes gained obligations to provide complex palliative care (as hospital admissions declined) while losing rights to specialist support (as specialist teams became remote consultants). Care staff were expected to manage symptoms, provide comfort care, and support families through death without the specialist training or support previously available. This obligation accumulation without corresponding rights expansion created situations where staff felt unprepared and unsupported in providing end-of-life care (42).

*Cascading Effects*: Rights erosion and obligation accumulation cascaded through multiple pathways. They created operational strain as positions were expected to do more with less. They generated moral injury as positioned actors felt unable to fulfil expanded obligations (26). They produced burnout as impossible demands accumulated (29). They eroded professional identity as care home staff felt reduced to crisis managers rather than care providers.

*Theoretical Significance*: This theme demonstrates how positioning is inherently political—it involves struggles over rights and obligations that reflect and reproduce power relations. Social positioning theory reveals that positions are not neutral structures but contested terrains where rights and obligations are negotiated. The pandemic involved repositioning systematically disadvantaged care homes while protecting governmental positions. Understanding this requires analysing not just current positional structures but processes through which positions are restructured during crises (21, 30, 31).

#### 3.2.4 Theme 4: information asymmetries and epistemic injustice

*Definition*: Systematic patterns where macro-level positions controlled information necessary for micro-level decision-making but failed to provide it, creating epistemic injustices where positioned actors were expected to make decisions without context relevant guidance. Positional Dynamics: Effective positioning requires that actors have information necessary to fulfil obligations. Across domains, care home positions were obligated to make critical decisions (accept admissions, implement isolation, allocate PPE, manage outbreaks) without information controlled by higher-level positions (infection status, supply availability, outbreak data, policy rationale). This created epistemic injustice—systematic disadvantage in knowledge production and distribution (32).

*Systems-Led Setbacks*: Information asymmetries represent a distinct category of systems-led setback: structural failures in knowledge systems that prevent positioned actors from fulfilling obligations. Testing unavailability meant care homes made admission decisions without infection status. Supply chain opacity meant care homes could not plan PPE procurement. Surveillance gaps meant outbreaks were detected late. These were not merely information gaps but systematic patterns where macro-level positions-controlled information micro-level positions needed but failed to provide it.

##### 3.2.4.1 Illustrative examples

*Testing and Surveillance*: Care homes were obligated to prevent infection entry and manage outbreaks without access to testing that would reveal infection status. This created a fundamental epistemic injustice: care homes were expected to control infection without knowing who was infected. Managers described making isolation decisions “in the dark,” implementing precautions based on symptoms alone despite evidence of asymptomatic transmission. The macro-level position (DHSC/PHE) controlled testing capacity but prioritised other settings, creating systematic information asymmetry (3).

*PPE Provision*: Care homes were obligated to procure adequate PPE without information about national supply levels, allocation criteria, or delivery timelines. This opacity forced care homes into unregulated markets without ability to distinguish legitimate suppliers from fraudsters. The macro-level position (DHSC) controlled supply information but did not share it, creating information asymmetry that left care homes vulnerable to exploitation (3).

*Cascading Effects*: Information asymmetries cascaded through multiple mechanisms. They created decision-making paralysis as actors lacked information needed for informed choices. They generated ineffective practises as decisions were based on incomplete information. They produced inequities as actors with better information access (often larger organisations with governmental connections) fared better. They eroded trust as information withholding was experienced as disrespect.

*Theoretical Significance*: This theme extends social positioning theory by highlighting epistemic dimensions of positioning. Positions are defined not only by rights and obligations but by epistemic rights—legitimate expectations of access to information necessary for fulfilling obligations. Information asymmetries represent epistemic injustice that undermines positioned agency. This connects social positioning theory to epistemic injustice literature, revealing how structural positioning shapes knowledge distribution (32). Fair positioning requires not only matching obligations with resources but ensuring epistemic rights—access to information necessary for informed action (3, 30, 31).

#### 3.2.5 Theme 5: temporal misalignments and structural lag

*Definition*: Systematic patterns where policy timelines at macro levels misaligned with operational realities at micro levels, creating temporal impossibilities where positioned actors were expected to implement changes faster than operationally feasible.

*Positional Dynamics*: Effective positioning requires temporal alignment between policy expectations and operational capacities. Across domains, macro-level positions set timelines based on policy priorities without accounting for implementation realities at micro levels. Discharge timelines (24–48 h) did not allow for infection control procedures (14-day isolation). Guidance changes occurred faster than care homes could implement them (3, 8). Vaccine delivery required coordination on timelines that did not match care home operational rhythms. These temporal misalignments created structural lag—systematic delays between policy expectations and operational capacity (17, 28).

*Systems-Led Setbacks*: Temporal misalignments represent a distinct systems-led setback: structural failures in temporal coordination that create impossible timelines. They reflect macro-level positions setting expectations without understanding micro-level operational realities. This is not merely poor planning but systematic pattern where powerful positions impose timelines on less powerful positions without consultation or consideration of feasibility.

##### 3.2.5.1 Illustrative examples

*Discharge Procedures*: Hospital discharge timelines (24–48 h from medical clearance) conflicted with infection control requirements (14-day isolation for potentially infected residents). This temporal impossibility forced care homes to choose between meeting discharge timelines (accepting residents immediately) or implementing infection control (isolating for 14 days). The macro-level position (NHS England) set discharge timelines based on hospital capacity needs without considering care home infection control requirements (13).

*Vaccine Rollout*: Vaccination teams often provided short notice (24–48 h) for vaccination clinics, requiring care homes to coordinate resident consent, family communication, and staff scheduling on impossible timelines. Frequent changes to prioritisation and delivery models meant care homes repeatedly replanned vaccination, with some changes announced after implementation had begun. This temporal chaos reflected macro-level policy flexibility without consideration of micro-level coordination requirements (3).

*Cascading Effects*: Temporal misalignments cascaded through multiple pathways. They created operational chaos as care homes scrambled to meet impossible timelines. They generated compliance failures as timelines could not be met. They produced inequities as organisations with more resources could adapt faster. They eroded trust as unrealistic timelines were experienced as disrespect for operational realities (31).

*Theoretical Significance*: This theme reveals temporal dimensions of positioning. Positions exist not only in social space but in time—they have characteristic temporal rhythms and coordination requirements. Temporal misalignments represent structural failures in temporal coordination. Social positioning theory must account for temporal dimensions: how positions are coordinated across time, how policy timelines align (or misalign) with operational rhythms, and how temporal power operates (who controls timelines, whose temporal needs are prioritised). This extends the theory by highlighting that fair positioning requires not only spatial coordination (rights, obligations, resources) but temporal coordination (feasible timelines, adequate notice, respect for operational rhythms) (13, 31, 33).

#### 3.2.6 Thematic integration

These five themes are interconnected, revealing how systems-led setbacks operated through multiple mechanisms simultaneously:

- Liminal positioning created vulnerability to systems-led setbacks.
- Accountability displacement obscured structural causes while blaming positioned actors.
- Rights erosion and obligation accumulation systematically disadvantaged care home positions.
- Information asymmetries prevented informed decision-making.
- Temporal misalignments created impossible timelines.

Together, these themes demonstrate how social positioning theory with systems-led setbacks illuminates the structural dynamics that shaped care home pandemic responses. The analysis reveals that failures were not primarily individual but structural—resulting from positional configurations that created impossible situations, systems-led setbacks that undermined capacity, and accountability systems that displaced responsibility downward while structural causes remained invisible.

**Figure 1:**
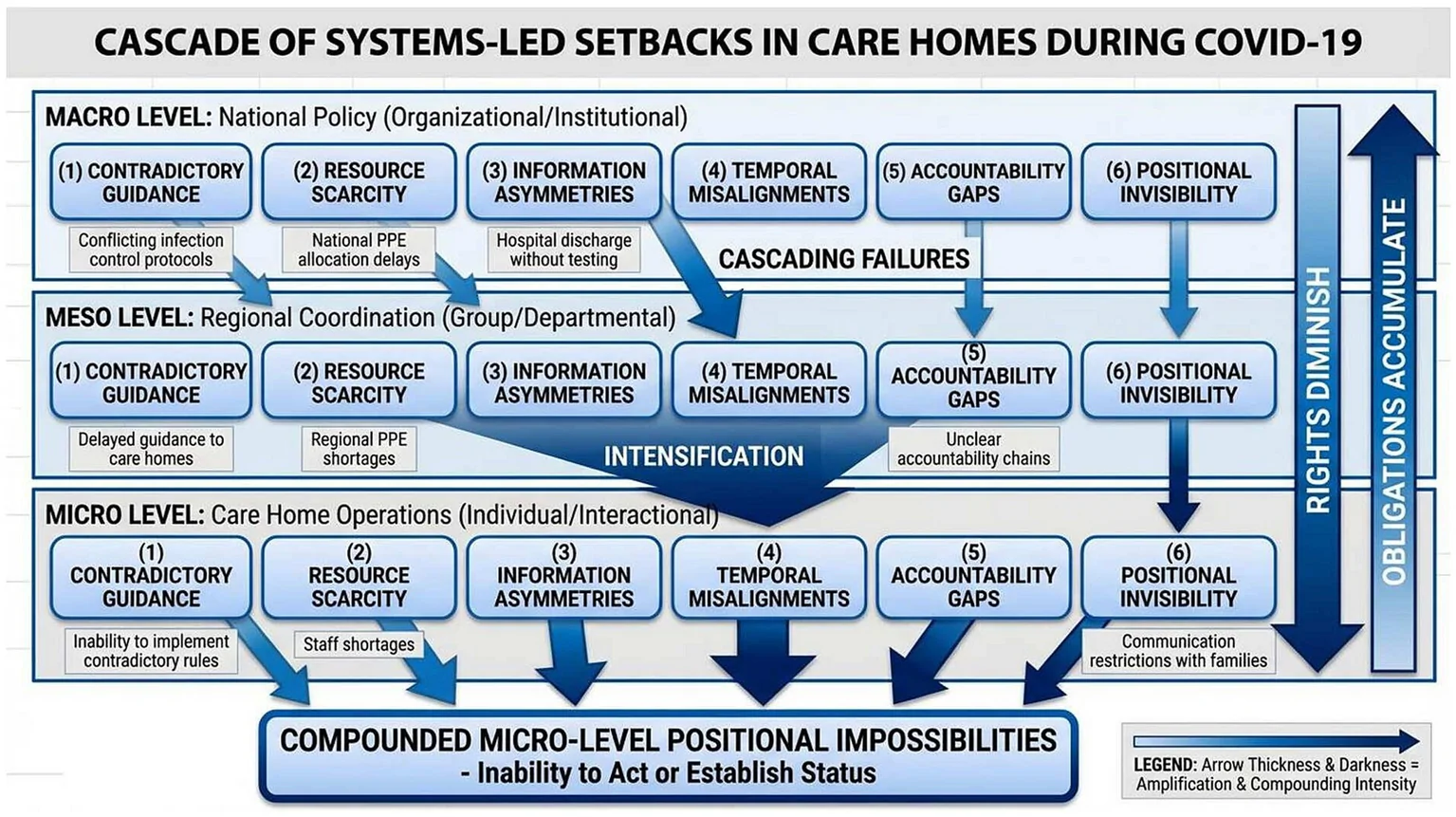
A multi-level cascade model showing how six systemic failure types propagated from macro (national policy) through meso (regional) to micro (care home) levels during COVID-19, compounding positional impossibilities, diminishing rights, and accumulating obligations for care home managers.

Figure 1 provides an overview of the main findings.

## 4 Discussion

### 4.1 Theoretical contributions

**Figure 2:**
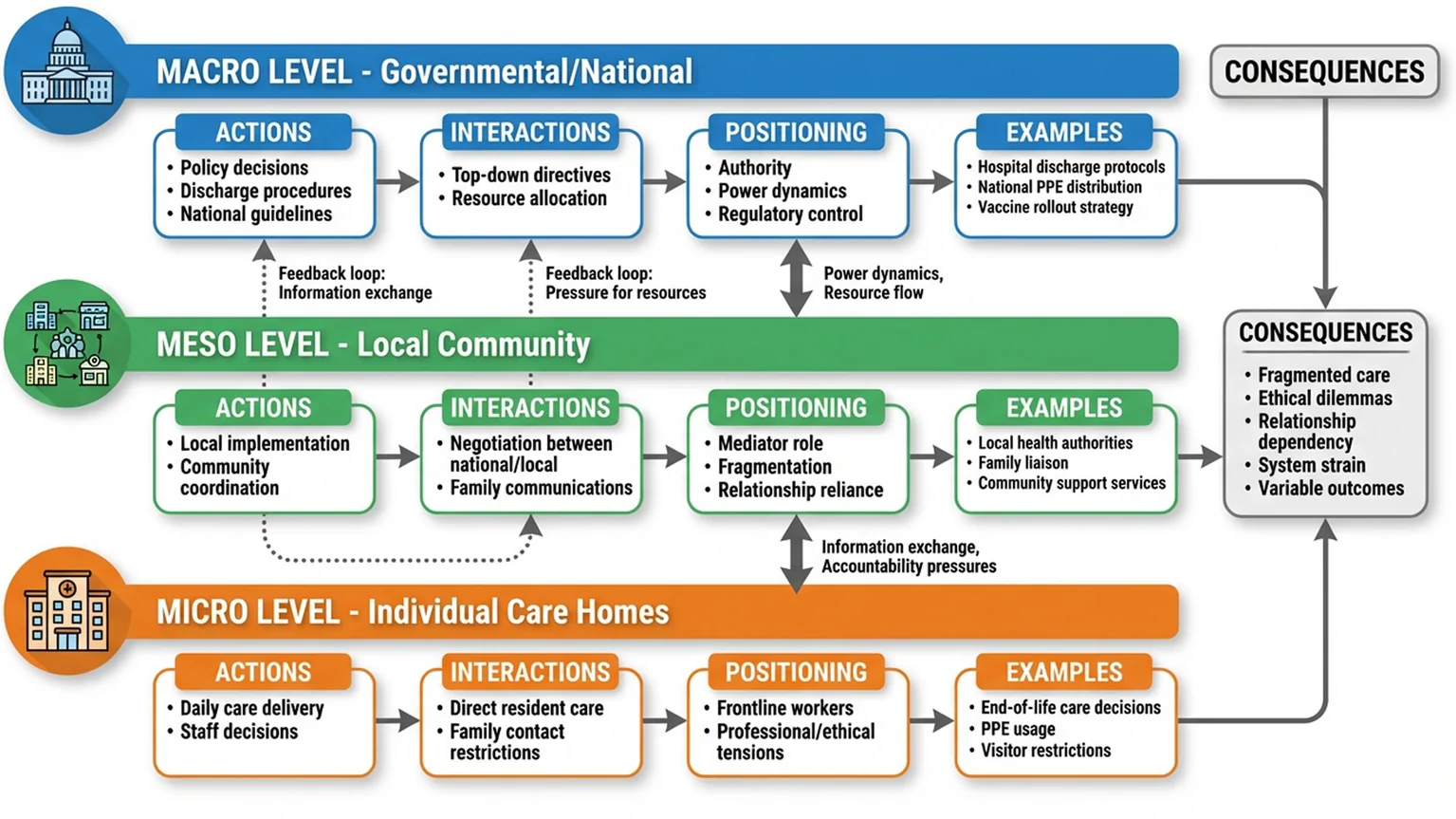
Thematic matrix mapping five analytical themes across six COVID-19 pandemic domains in English care homes.

This analysis makes several contributions to social positioning theory and its application in healthcare contexts. The integration of systems-led setbacks extends social positioning theory by providing analytical tools for understanding structural failures. While Slade-Caffarel’s framework analyses how positions, rights, obligations, and practises are structured, systems-led setbacks reveal how these structures can systematically fail. This extension maintains the theory’s core insights while adding explanatory power for understanding crises and structural injustices (Figure 2).

The analysis demonstrates how positions at different organisational levels (macro, meso, micro) interact through hierarchical relationships where higher-level positions shape conditions for lower-level positions. This multi-level analysis reveals cascading effects—how failures at higher levels propagate downward, often amplifying rather than buffering impacts. Understanding healthcare systems requires analysing not just positions at single levels but inter-level dynamics (28, 34).

The analysis also reveals systematic patterns where rights and obligations become misaligned—positions gain obligations without corresponding rights or lose rights while retaining obligations. This asymmetry creates positional vulnerabilities that systems-led setbacks exploit. Social positioning theory must attend to rights-obligations balance as a criterion for fair positioning (35).

The theme of information asymmetries extends social positioning theory by highlighting epistemic rights—legitimate expectations of access to information necessary for fulfilling obligations. This connects social positioning theory to epistemic injustice literature, revealing how structural positioning shapes knowledge distribution (32). Positions are defined not only by material rights and obligations but epistemic rights and obligations.

The theme of temporal misalignments reveals that positions exist in time as well as social space. Positions have characteristic temporal rhythms, coordination requirements, and timeline expectations. Temporal power—who controls timelines, whose temporal needs are prioritised—represents an undertheorized dimension of positioning. Fair positioning requires temporal coordination as well as spatial coordination (33, 43, 44).

### 4.2 Connections to existing literature

This analysis connects social positioning theory to several established literatures.

The concept of liminal positioning creating impossible obligations connects to literature on moral injury in healthcare—psychological distress resulting from perpetrating, failing to prevent, or bearing witness to acts that transgress moral beliefs (26). Systems-led setbacks create conditions for moral injury by forcing positioned actors into situations where all available actions violate moral obligations. This suggests that addressing moral injury requires structural reforms, not merely individual resilience training.

The analysis resonates with Lipsky’s street-level bureaucracy theory, which examines how frontline public service workers exercise discretion under conditions of resource scarcity and policy ambiguity (30). Social positioning theory extends this by analysing how positions are structured through rights and obligations, revealing that “discretion” often means choosing between impossible alternatives created by systems-led setbacks. The analysis suggests that street-level workers’ challenges result not from excessive discretion but from liminal positioning.

The theme of accountability displacement connects to organisational justice literature examining fairness in blame attribution and accountability systems (36). The analysis reveals that accountability systems often invert causal chains, holding lower-level positions accountable for failures caused by higher-level structural conditions. Fair accountability requires tracing failures to structural origins rather than stopping at most visible actors.

The theme of information asymmetries connects to Fricker’s epistemic injustice framework, which examines systematic disadvantages in knowledge production and distribution (32). Social positioning theory reveals how structural positioning creates epistemic injustices—positioned actors expected to make decisions without information controlled by higher-level positions. This suggests that epistemic justice requires not only testimonial and hermeneutical justice but structural reforms ensuring epistemic rights.

### 4.3 Implications for healthcare system design

The analysis has several implications for healthcare system design.

*Position-Capacity Alignment*: Healthcare systems must ensure that positional obligations are matched with enabling rights, resources, and information. Liminal positioning—high obligations with limited rights—creates systematic vulnerabilities. System design should audit positions for rights-obligations balance and address misalignments (33, 44).

*Cascading Effect Prevention*: Multi-level systems must include buffering mechanisms that prevent failures at higher levels from cascading to lower levels. Currently, organisational hierarchies often amplify rather than buffer impacts. System design should include redundancy, flexibility, and support mechanisms that absorb shocks rather than transmitting them downward.

*Accountability Alignment with Causation*: Accountability systems must align with causal responsibility—holding positions accountable for outcomes they have power to influence while recognising structural constraints. Current systems often displace accountability downward while structural causes remain unaddressed. Fair accountability requires tracing failures to structural origins (33, 43, 44).

*Epistemic Infrastructure*: Healthcare systems must ensure that positioned actors have access to information necessary for fulfilling obligations. This requires epistemic infrastructure—testing systems, surveillance networks, information sharing protocols—that distribute knowledge equitably rather than concentrating it at higher levels (32).

*Temporal Coordination*: Policy timelines must align with operational realities. Macro-level positions must consult micro-level positions when setting timelines, ensuring feasibility rather than imposing expectations based solely on policy priorities. Temporal coordination requires respecting operational rhythms and providing adequate notice for changes (33, 43, 44).

### 4.4 Limitations and future directions

This analysis has several limitations that suggest future research directions:

*Document-Based Analysis*: The study analyses participant accounts and policy documents about care home responses. It was not possible to include the direct accounts of those tasked with cascading policy into practise at national and local level organisations such as Local Authorities or NHS. Future research should examine lived experiences of positioned actors, exploring how structural positioning is experienced and navigated in practise, this may be difficult to achieve during a crisis because of resource and access permissions.

*Single Context Focus*: The analysis focuses on UK care homes during COVID-19. Future research should examine whether similar positional dynamics and systems-led setbacks operate in other healthcare contexts, countries, and crises, testing the framework’s generalizability. Comparisons with other countries would widen understanding. It likely that aspects of these findings will resonate with other countries where population demographics are similar and care for older people leans towards care home and domicillary care provision in the community. Parrallels with achieving parity of resource supplies compared to urgent hospital care and distinct knowledge of the care home sectors in other countries may also resonate with these findings and analysis.

*Retrospective Analysis*: These theoretical analyses pandemic policy and practise responses retrospectively combined with data obtained in real time from care home managers during the pandemic. Future research should examine positioning dynamics in real-time during crises, capturing how positions emerge and are contested as events unfold.

*Limited Attention to Resistance*: The analysis focuses on how structural positioning constrained action but gives less attention to how positioned actors resisted, subverted, or transformed positions. Future research should examine positioned agency—how actors navigate, contest, and reshape structural positions (41, 43).

*Intervention Research Needed*: This analysis is descriptive and critical. Future research should develop and test interventions based on social positioning theory—structural reforms designed to align rights and obligations, prevent cascading effects, and ensure fair accountability.

### 4.5 Implications for theory and practise

#### 4.5.1 Theoretical implications

*Social Positioning Theory Development*: This analysis demonstrates social positioning theory’s utility for analysing complex healthcare crises while extending the framework through systems-led setbacks, epistemic dimensions, and temporal dimensions. Future theoretical work should further develop these extensions, examining how they apply across contexts and integrate with the theory’s core concepts (33, 43, 44).

*Integration with Critical Theories*: Social positioning theory connects productively with critical theories examining power, injustice, and structural violence. The framework provides analytical tools for making structural causation visible while preserving space for positioned agency. Future work should explore connections with feminist theory, critical race theory, and disability studies, examining how positioning intersects with other forms of structural disadvantage.

*Methodological Development*: This study demonstrates thematic analysis as a method for applying social positioning theory. Future methodological work should develop additional approaches—ethnographic methods for observing positioning in practise, participatory methods for involving positioned actors in analysis, and intervention methods for testing structural reforms based on positioning theory (33, 43, 44).

#### 4.5.2 Policy implications

*Structural Audit of Healthcare Positions*: Policymakers should conduct systematic audits of healthcare positions, examining rights-obligations balance, resource adequacy, information access, and temporal feasibility. Positions with high obligations but limited rights require structural reform to prevent liminal positioning (33, 43, 44).

*Cascading Effect Assessment*: Policy proposals should include cascading effect assessments—systematic analysis of how policies impact positions at different organisational levels. Policies that create benefits at higher levels while imposing costs at lower levels require redesign or compensatory support mechanisms.

*Accountability System Reform*: Accountability systems should be reformed to align with causal responsibility. This requires tracing failures to structural origins rather than stopping at most visible actors and holding macro-level positions accountable for providing enabling conditions for micro-level positions (33, 43, 44).

*Epistemic Infrastructure Investment*: Healthcare systems require investment in epistemic infrastructure—testing, surveillance, and information systems—that ensures positioned actors have information necessary for fulfilling obligations. Information should be distributed equitably rather than concentrated at higher levels (32).

*Temporal Coordination Mechanisms*: Policy development should include consultation with frontline positions about timeline feasibility. Macro-level positions should provide adequate notice for changes and respect operational rhythms rather than imposing timelines based solely on policy priorities (33, 43, 44).

#### 4.5.3 Practise implications

*Positional Awareness*: Healthcare managers and frontline workers should develop positional awareness—understanding their own positions’ rights, obligations, and structural constraints, and recognising how their positions relate to others. This awareness can inform advocacy for structural reforms including parity with the NHS and realistic expectations about what positioned actors can achieve (27).

*Collective Advocacy*: Positioned actors facing liminal positioning and systems-led setbacks should engage in collective advocacy for structural reforms. Individual coping strategies are insufficient when problems are structural. Professional organisations, unions, and advocacy groups should use evidenced-based research, theory and lived experiences to articulate structural problems and demand robust systemic solutions (35, 37, 38).

*Documentation of Systems-Led Setbacks*: Frontline workers should document systems-led setbacks—recording when structural failures prevent fulfilling obligations. This documentation can make structural causation visible, countering accountability displacement and supporting advocacy for reforms.

*Moral Injury Prevention*: Healthcare organisations should recognise that moral injury results from structural positioning, not individual weakness. Prevention requires structural reforms that eliminate impossible situations, not merely resilience training. Organisations should audit positions for moral injury risks and address structural causes (26).

## 5 Conclusion

The COVID-19 pandemic exposed profound vulnerabilities within the relationship between health and social care systems. Through analysis of care-home managers’ experiences, this study demonstrates how uncertainty shocks generated systems-led setbacks across multiple domains of pandemic response. These setbacks emerged not simply from operational failures but from interactions between organisational positioning, accountability structures, information flows, and resource dependencies.

Social positioning theory provides a valuable framework for understanding why care homes experienced these challenges so acutely. Managers occupied positions characterised by expanding responsibilities but constrained authority, resources, and access to information. Under conditions of uncertainty, these positional vulnerabilities amplified the organisational consequences of policy and system-level decisions.

The findings suggest that future preparedness efforts should focus not only on strengthening resources and infrastructure but also on addressing the organisational relationships through which uncertainty is distributed and managed. Greater attention to positioning, information governance, organisational inclusion and system integration may help reduce the likelihood of systems-led setbacks during future crises and strengthen the resilience of health and social care systems more broadly.

## Data Availability

The datasets presented in this article are not readily available because potentially identifiable data by geography and institution despite anonymisation. Requests to access the datasets should be directed to Adam.gordon@qmul.ac.uk.

## Author’s note

This paper is dedicated to all those care home residents, including my mum, June Marshall, who died of COVID-19.
