# Supplementary material for: Leading through crisis: care home managers’ COVID-19 experiences examined through systems-led setbacks and social positioning theory
Source: Front Public Health. 2026 Sep 10;14:1916166. doi: 10.3389/fpubh.2026.1916166 (PMC13602212; doi:10.3389/fpubh.2026.1916166)
Supplement: Supplementary file 1 [file Supplementary_file_1.docx]

**Master Policy Timeline**

*Care Home COVID-19 Research Project — Cross-Cutting Chronological Summary*

This table collates all policy dates referenced across the six thematic source documents of this project into a single chronological record. Each entry is drawn directly from the policy timeline column of the relevant document; no dates, facts, or references have been added or inferred. Three themes — PPE Supplies and Education, Discharge Procedures and Hospital Discharge, and Provision of End-of-Life Care — contained no specific policy dates in the source material and are therefore not represented in the timeline below; this is noted at the foot of the table.

| **Date** | **Theme** | **Level** | **Policy Development** |
| --- | --- | --- | --- |
| **March 2020** | Testing for Covid Infection | *Macro Level* | Care home testing ceased. |
| **April 2020** | Testing for Covid Infection | *Macro Level* | Asymptomatic testing introduced. |
| **8 June 2020** | Testing for Covid Infection | *Macro Level* | Mandatory testing enacted; self-isolation support payment introduced. |
| **July 2020** | Testing for Covid Infection | *Macro Level* | Research programme across 100 care homes initiated. |
| **July 2020** | Family Communications and Visits | *Macro Level* | Multiple iterations of visiting guidance issued, incorporating outdoor visits, window communications, a single-visitor limit, and mandatory PPE use. |
| **November 2020** | Vaccine Roll-out | *Meso Level* | Lateral flow tests distributed to local authorities. |
| **November 2020** | Testing for Covid Infection | *Macro Level* | Lateral flow tests distributed to local authorities. |
| **November 2020** | Testing for Covid Infection | *Meso Level* | Lateral flow tests sent to local authorities; access variable for smaller and rural homes. |
| **8 December 2020** | Vaccine Roll-out | *Macro Level* | Fifty hospital hub vaccination centres established across the UK. |
| **8 December 2020** | Vaccine Roll-out | *Meso Level* | Physical access to hospital hub vaccination sites for care home residents confirmed as minimal. |
| **December 2020** | Testing for Covid Infection | *Consequences* | Family visitor testing requirement introduced, generating staff–family tensions. |
| **Late December 2020** | Testing for Covid Infection | *Macro Level* | Lateral flow tests confirmed as accurate for use in care home settings (subject to ongoing academic debate into 2021). |
| **30 December 2020** | Vaccine Roll-out | *Macro Level* | Care home residents prioritised for vaccination; vaccination mandated for all care home staff (no equivalent mandate for NHS staff). |
| **January 2021** | Family Communications and Visits | *Micro Level* | Key relative ('essential caregiver') scheme recommended; discretionary application noted, producing inconsistency across the sector. |
| **2021** | Family Communications and Visits | *Macro Level* | Designated 'essential caregiver' role formally introduced, permitting sustained access by a named relative. |
| **Until 2022** | Family Communications and Visits | *Micro Level* | Total visit bans persisting in some care homes (Dixon, J et al., 2023**). |

**Colour key**

**Blue rows:** Testing for Covid Infection

**Green rows:** Vaccine Roll-out from Early December 2020

**Amber rows:** Family Communications and Visits

** Authorial note: All entries are drawn exclusively from the Policy Timeline columns of the six thematic documents produced for this project. No additional dates, citations, or external sources have been introduced. Three themes contained no specific policy dates in the source material and are therefore absent from this timeline: (1) PPE Supplies and Education; (2) Discharge Procedures, Testing and Hospital Discharge; (3) Provision of End-of-Life Care. ** The reference (Dixon, J et al., 2023) appeared in the original source material for Family Communications and Visits; it is retained as supplied and has not been independently verified.*
